# Monitoring Prescribing for Attention Deficit Hyperactivity Disorder (ADHD) in Child & Adolescent Mental Health Service (CAMHS): A Clinical Audit

**DOI:** 10.1192/bjo.2026.11359

**Published:** 2026-06-30

**Authors:** Yu Ching Riva Cheng, Mercedes Smith, Christabel Boyle

**Affiliations:** 1 University of Glasgow, Glasgow, United Kingdom; 2 NHS Scotland, Glasgow, United Kingdom

## Abstract

**Aims:**

ADHD is treated in CAMHS and may involve non-pharmacological treatments, suchas behavioural therapy. It may also involve pharmacological treatments, such as methylphenidate, lisdexamfetamine, atomoxetine, and guanfacine. Medication side effects include hypertension, weight loss, arrhythmias, and loss of appetite, all of which are detrimental to childhood development. The aim of this closed-loop audit was to improve Attention Deficit Hyperactivity Disorder (ADHD) monitoring within the Child & Adolescent Mental Health Services (CAMHS) in West Glasgow. Patients’ height, weight, blood pressure, and heart rate, along with respective centiles and growth charts, were assessed in accordance with NICE guidelines.

**Methods:**

In the initial audit, a retrospective data search using electronic health records identified a sample of 33 patients under 18 with a diagnosis of ADHD who took ADHD medication within the previous 6 months. The results of this initial audit were presented at a local department meeting. Posters of the NICE guidelines were then distributed. The same methods as the initial audit were used to identify 45 patients on ADHD medication for the follow-up cycle to assess improvement in monitoring.

**Results:**

In the initial audit, 97% of the 33 patients had their height and weight recorded, and 100% had their blood pressure and heart rate recorded. 85% of patients had their height centile, weight centile, and growth charts recorded. 10% of patients had blood pressure centiles recorded, and 0% had heart rate centiles recorded. In the second cycle, 100% of the 45 patients had their height, height centiles, weight, weight centiles, heart rate, blood pressure, and growth charts recorded. 2% of patients had blood pressure centiles recorded, and 0% had heart rate centiles recorded.

**Conclusion:**

There was a significant increase in the monitoring of height, height centiles,weight, weight centiles, heart rate, blood pressure, and growth charts in the second cycle compared with the first cycle. However, blood pressure and heart rate centiles still did not meet NICE guidelines. This was clinically significant due to the variance in heart rate and blood pressure in children. The electronic health system’s inability to convert raw figures of blood pressure and heart rate to percentiles likely impacted the ability to monitor these specific percentiles.

